# Rice Adaptation to Abiotic Stresses Caused by Soil Inorganic Elements

**DOI:** 10.3390/ijms26157116

**Published:** 2025-07-23

**Authors:** Giulia Vitiello, Daniela Goretti, Caterina Marè, Edoardo Delmastro, Giorgia Siviero, Silvio Collani, Erica Mica, Giampiero Valè

**Affiliations:** 1 Department for Sustainable Development and Ecological Transition, University of Eastern Piedmont, Piazza Sant ’Eusebio 5, 13100 Vercelli, Italy; daniela.goretti@uniupo.it (D.G.); edoardo.delmastro@uniupo.it (E.D.); giorgia.siviero@uniupo.it (G.S.); silvio.collani@uniupo.it (S.C.); erica.mica@uniupo.it (E.M.); giampiero.vale@uniupo.it (G.V.); 2 Research Centre for Genomics and Bioinformatics, Council for Agricultural Research and Economics (CREA), 29017 Fiorenzuola d’Arda, Italy; caterina.mare@crea.gov.it

**Keywords:** rice, toxic elements, stress, tolerance, genetic resources, breeding, genome editing, microbiota

## Abstract

Soil contamination with toxic inorganic elements poses a major challenge to rice cultivation, affecting plant physiology, yield, and grain safety. While natural variation in tolerance exists among rice genotypes and related species, recent advances in genomics, breeding, and biotechnology offer new opportunities to enhance adaptation. This review synthesizes the current knowledge on the physiological effects of toxic elements and explores strategies to improve tolerance, from harnessing genetic diversity to genome editing and transgenic approaches. Attention is also paid to the role of microbiota in mitigating toxicity and reducing translocation to seeds, highlighting emerging solutions for sustainable rice production in contaminated environments.

## 1. Introduction

Soil is a complex and dynamic ecosystem, essential as it supports food production and maintains water quality. Soil consumption due to pollution, erosion, and the increase in urbanized and industrial areas is decreasing the amount of land available for agricultural use. Moreover, arable lands can no longer be expanded in order to preserve natural areas, which are a valuable source of biodiversity. One of the major global challenges nowadays is to maintain healthy and productive soils to ensure food security for a growing population (expected to reach 9.1 billion by 2050) [1], while simultaneously reducing the environmental impact of agriculture, including its contribution to climate change. Sustainable agriculture has to provide enough healthy food to contrast both food insecurity and “hidden hunger”, i.e., a scarcity of micronutrients and insufficient caloric intake [2]. Among various factors influencing crop yield, food quality, and human health, field contamination by heavy metals (HMs) and salinization stands out as a major cause diminishing agricultural soil quality and productivity [3,4]. This is particularly relevant, as 19.5% of irrigated agricultural soils are considered saline, having an electrical conductivity above 4 dS m^−1^ [5,6], due to fertilizer overuse and poor water quality [7,8], or because of saline water infiltration in coastal areas, a phenomenon exacerbated by climate change. There is no definitive percentage for HMs in agricultural soil globally. Anyway, in China, almost 20% of agricultural soil is estimated to be HMs- contaminated [9]. HMs are defined as having a specific density greater than 5 g/cm^3^ and are usually divided into elements that are useful to living organisms (such as copper (Cu), boron (B), zinc (Zn), iron (Fe), selenium (Se), molybdenum (Mo), and manganese (Mn)) and those that are detrimental (such as Arsenic (As), cadmium (Cd), lead (Pb), mercury (Hg), thallium (Tl), and chromium (Cr)). Both classes of elements are considered toxic and harmful to plants when present above certain thresholds, inhibiting cellular growth and disrupting normal homeostasis. Soil and water HM contamination is predominantly attributed to anthropogenic activities, in particular, mining operations and metal smelting, as they release substantial quantities of toxic metals into the surrounding environment. Additionally, the widespread historical use of Pb gasoline has significantly increased its concentration in both terrestrial and aquatic ecosystems. Agricultural practices also play a critical role, particularly through the application of agrochemicals containing As- or Hg-based compounds. Furthermore, contamination can be exacerbated by the use of animal manure derived from livestock fed with mineral-enriched commercial feed, which introduces additional HMs into agricultural soils. These sources, often acting in combination, result in long-term environmental contamination and pose serious risks to human health and ecological systems [10,11]. Salinity and HM pollution in agricultural soils have direct effects on the soil ecosystem itself, reducing fertility and altering microbial biodiversity, and indirectly negatively affecting crops. Sodic soils have unfavorable physic properties, are poorly aerated, and have low water infiltration, hindering nutrient cycling [12]. An excessive concentration of Na^+^ and Cl^−^ in soil increases cellular osmotic pressure [13], decreases photosynthetic efficiency, disrupts intracellular reactive oxygen species (ROS) balance [14,15], and reduces plant nutrient uptake, inhibiting root growth [16,17]. Similarly, plants growing in HM-contaminated soils show visible symptoms, like reduced growth, chlorosis, root browning, and an alteration in root morphology [18]. A high HM concentration in leaves might hamper photosynthesis interfering with electron transport chain (ETC)and enzyme activity, in the end reducing plant yield. Soil chemical properties, such as pH, salinity, and aerobic/anaerobic conditions, might strongly influence the solubility and bioavailability of such elements. For example, a low pH, caused by excessive N fertilization, increases the solubility and bioavailability of cationic metals (Cd and Pb), promoting their uptake by plants and subsequent entry into the food chain [19]. High salinity was shown to also promote the mobilization of Cd, Cu, and Pb [20,21]. Aerobic conditions favor Cd bioavailability and accumulation in plants, while anoxic conditions, typical in paddy submerged soils, favor arsenate As(V) reduction to arsenite As(III), which is more bioavailable to rice roots [22,23]. Rice (*Oryza sativa*), as a staple food for more than half of the global population, is one of the most important dietary source of HMs, especially As, Cd, and Hg, as it accumulates higher levels of HMs in its edible part when compared to other cereals, like wheat and maize [24,25]. This makes its management in contaminated areas particularly challenging, as rice cultivation practices, such as prolonged flooding, promote ion solubilization. This poses an important concern for human health and must be addressed when it comes to rice farming. Furthermore, rice is the most susceptible cereal to soil salinization, causing an average annual yield reduction in the range of 30–50% [26]. Rice polishing, which involves the removal of the outer layers of rice kernels to obtain white rice, can reduce HM kernel levels depending on the specific element [27]. As and Hg are mostly present in the outer layers of the kernel, while their organic methylated forms can translocate to the endosperm [28,29]. In contrast, Cd shows a less pronounced preference for accumulation in the outer tissues [30], while Pb is completely removed with brown rice polishing as it is retained in the bran layer [31]. Reducing contaminant accumulation in rice therefore requires an integrated approach, combining agronomic strategies from a sustainable agriculture perspective (e.g., irrigation management, application of soil conditioners), genetic improvement, and biotechnological and microbiological interventions. In particular, the use of unconventional genetic resources, such as local and traditional varieties and wild relatives of *O. sativa*, has enabled the identification of loci and genes associated with tolerance, providing valuable tools for varietal improvement. The integration of molecular breeding techniques, including genome-wide genotypic association studies (GWAS) and marker-assisted selection (MAS), has already produced promising results in international programs. Along with these approaches, increasing attention is being paid to the role of rhizosphere- and endosphere-associated microbiota, which can modulate metal bioavailability, enhance selective nutrient uptake, and stimulate plant defense mechanisms. This review offers an integrated and updated perspective on the physiological and molecular responses of rice to toxic elements, emphasizing the significance of genetic diversity in enhancing tolerance. The paper offers an original and comprehensive examination of HMs and salt stress, two interconnected phenomena rarely addressed together. It also underscores the crucial yet under-explored role of soil microbiota in enhancing plant resilience to salt and metal stress, as well as its role in regulating the bioavailability of toxic elements to plants. These findings contribute to the current body of knowledge on abiotic stress resistance and rice seed safety.

## 2. Physiological Effects of Toxic Elements

**Figure 1:**
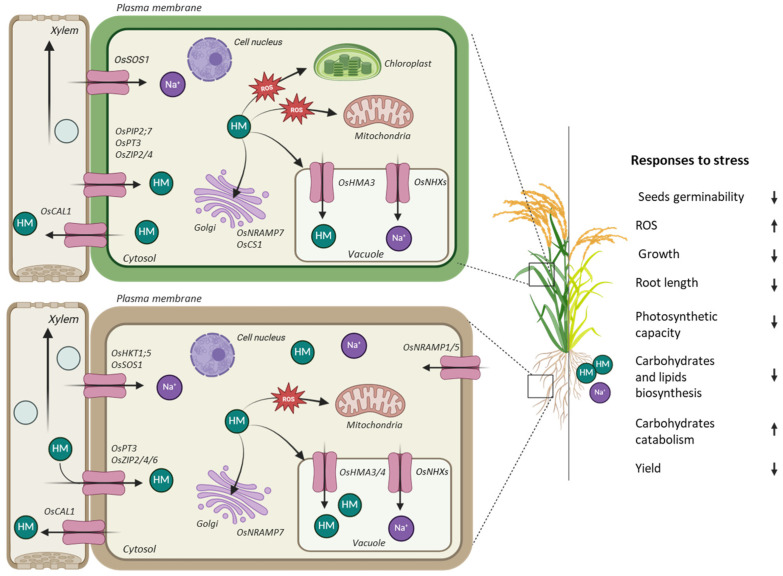
Physiological mechanisms of rice in response to heavy metal and salt stress. On the left: key cellular responses, including the activation of specific transporters and ion compartmentalization within leaf (top panel) and root (bottom panel) cells. Vacuolar sequestration mechanisms are highlighted, with representative transporters involved in detoxification. On the right: arrows indicate the upward or downward trend in the parameters involved in stress response.

HMs, such as Cd, As, Pb, Hg, and Cr, and high salt concentrations in the environment pose major challenges to human health and rice production, affecting physiological and morphological traits, including germination, growth, and yield. Given the wide range of effects of HMs on plant development, a comprehensive understanding of their uptake mechanisms, transport pathways, and implications for human health and crop management and productivity is essential. Schematic information is summarized in Figure 1.

The accumulation of reactive oxygen species (ROS) is a prototypical response exhibited by plants as a reaction to both of these abiotic stresses. It instigates intricate signaling cascades that activate gene regulatory networks in an attempt to counteract oxidative damage and restore cellular homeostasis. As a fundamental component of these networks, transcription factors (TFs), such as NAC, WRKY, and bZIP, act as master regulators of antioxidant defense. NAC TFs have been demonstrated to regulate the expression of ROS-scavenging enzymes, including superoxide dismutase (SOD), catalase (CAT), and ascorbate peroxidase (APX), and to modulate downstream stress-responsive pathways. For instance, *OsNAC5* is induced by hydrogen peroxide (H_2_O_2_) triggered by Cd exposure, upregulating genes involved in Cd tolerance [32], while *SNAC3* confers As tolerance by mitigating oxidative stress [33]. In a similar manner, *OsNAC45* exerts its influence over salt sensitivity through the modulation of ROS levels [34]. WRKY TFs have been observed to bind to W-box elements in antioxidant gene promoters, thereby integrating ROS and hormonal signaling, particularly salicylic acid and abscisic acid. Recent reviews have emphasized the role of WRKY in mediating tolerance to HMs and salinity [35,36]. In addition to their other functions, bZIP TFs have been shown also to contribute to the regulation of antioxidant genes. Furthermore, they have been observed to interact with the ABA and MAPK pathways, thereby enhancing the detoxification of ROS. While the role of bZIP18 and bZIP32 in HM tolerance remains to be fully characterized, the evidence suggests a potential involvement in redox regulation [37]. In contrast, the function of these genes in salt stress is better established, with *OsZIP71* [38], *OsHBP1* [39], and *OsbZIP62* [40] promoting salt tolerance. Collectively, these TFs orchestrate a series of enzymatic and non-enzymatic antioxidant responses, thereby maintaining redox homeostasis under stress.

ROS generation overwhelms plant antioxidant defenses, disrupts cellular homeostasis, and damages critical biomolecules, and has been observed in chloroplasts, mitochondria, and peroxisomes as primary sites (Figure 1). In chloroplasts, HMs were reported to disrupt the ETC in photosystems I and II, causing electron leakage and the subsequent reduction of oxygen to superoxide anions [41]. Peroxisomal β-oxidation of fatty acids and photorespiration were identified as additional sources of ROS. These processes are enhanced by HMs through lipid peroxidation, increasing H_2_O_2_ production [42]. Roots, as primary uptake site, are particularly affected by HMs. Cd and Pb, for example, have been shown to reduce root elongation and thickening due to the inhibition of cell division in the apical meristem [43,44]. Decreased root hair length was also observed, further impairing nutrient and water absorption [45]. In aerial parts, HM stress leads to leaf chlorosis, necrosis, a reduced panicle size, and a lower grain yield due to impaired chlorophyll synthesis and spikelet formation [46,47]. Additionally, grains are often reduced in size and weight, diminishing their nutritional value [48]. The uptake of HMs in rice is primarily mediated by metal transporters in root cells. For example, Cd uptake often occurs through the low-affinity cation transporter *OsNramp*5, which also facilitates the uptake of essential nutrients, like Mn [49]. Similarly, As is absorbed by the roots as arsenite via aquaporins (such as *OsLsi*1) or as arsenate via phosphate transporters (*OsPT*1 and *OsPT*8), due to its chemical similarity [50,51]. Once absorbed, these metals are transported to aerial parts via the xylem. In root cells, metals like Cd and inorganic As are chelated with phytochelatins, thiol-rich peptides, and these complexes are sequestered into vacuoles, restricting translocation to the shoots and grains [52,53]. However, in certain genotypes, Cd was observed to bypass sequestration mechanisms and accumulate in rice grains, posing dietary risk [53]. As often accumulates in grains in organic forms, such as dimethyl arsenic acid, which originates from the microbial methylation in flooded paddy soils [52]. Uptake and grain accumulation of Cd and As in rice are significantly influenced both by soil bioavailability and rice cultivar, with relevant impacts also on seeds germination, interfering with water uptake and enzymatic activities [54]. In seeds, Cd inhibits amylase, peptidase, and protease enzyme activities, essential for breaking down stored nutrients [55,56]. The reduction in enzymatic activity leads to delayed or incomplete germination. Similarly, As affects seed viability by inducing oxidative stress, which damages cellular structures and impairs energy metabolism [57]. Exposure to Cu and As has been shown to cause intergenerational effects, reducing F1 seeds’ germination from metal-treated F0 plants [58]. Similarly, Pb and Hg have shown reduced rice germination index values [59]. HMs affect both the structure and function of photosynthetic machinery, first inhibiting chlorophyll biosynthesis. Cd and Pb interfere with the uptake of Mg and Fe, which are critical for chlorophyll production, causing chlorosis and reducing the photosynthetic capacity [60]. Moreover, Cd exposure reduces photosystem II activity, impairing light energy capture and electron transport, decreasing the photosynthetic rate, carbon assimilation, and therefore plant growth and productivity [61]. Some metals, like As, reduce stomatal opening to limit water loss, restricting CO_2_ uptake and further hindering photosynthesis [62]. Lipid and carbohydrate metabolism are compromised through lipid peroxidation and disrupted carbon assimilation. ROS, generated under stress, attack membranes and polyunsaturated fatty acids, forming malondialdehyde (MDA), and compromising membrane integrity [63]. HMs also impair lipid biosynthesis, especially phospholipids and glycolipids, and carbohydrate biosynthesis interfering with sucrose synthase and invertase, exacerbating photosynthetic and cellular dysfunction [64,65]. This influences sucrose partitioning between source (leaves) and sink (grains) tissues, affecting grain filling and yield. HM stress also reduces the activity of starch synthases, leading to decreased starch accumulation in rice grains [66]. At the same time, HMs can trigger the activation of catabolic pathways, increasing the breakdown of stored carbohydrates to provide energy for stress responses. HMs also disrupt nutrient uptake, interfering with essential elements, like N, P, K, Ca, Mg, Fe, and Zn. Cd competes with Mn, Ca, and Zn via transporters like *OsNramp*5 and *OsHMA*3 [67,68]. As impairs P uptake by competing with phosphate ions [69]. Additionally, HMs inhibit nitrate reductase activity, reducing protein production [70]. Exposure to HMs not only impairs physiological functions, including photosynthesis and enzyme activity, but also compromises rice tolerance to abiotic and biotic stresses. For example, reduced Zn levels increase susceptibility to oxidative stress and disease. Mitigation strategies, such as soil amendments, biofortification, and the use of metal-tolerant rice varieties, are essential to restore nutrient balance and ensure sustainable rice production in contaminated areas.

Rice productivity is significantly affected by salinity stress, mainly caused by sodium chloride (NaCl) in the soil. Understanding the mechanisms of NaCl uptake and transport in rice is crucial for developing salt-tolerant cultivars. Na^+^ uptake in rice predominantly occurs through two main pathways: the apoplastic pathway and the symplastic pathway. The apoplastic pathway involves an unregulated and passive movement of Na^+^ through the cell walls and intercellular spaces driven by diffusion, contributing significantly to Na^+^ accumulation, especially under saline conditions [71]. The symplastic pathway, in contrast, involves the active transport of Na^+^ across the plasma membrane into the cytoplasm. Recent studies have found that this process is mediated by specific ion transporters, including high-affinity potassium transporters (HKTs) and non-selective cation channels (NSCCs). Among them, *OsHKT1;5* has shown an important role reducing Na^+^ transport to the shoots by retrieving Na^+^ from the xylem, thereby enhancing salt tolerance [72,73]. Once inside the plant, Na^+^ is distributed between root and shoot tissues. An efficient regulation of this process could be a key to minimizing cellular toxicity. In rice, *OsSOS1*, a plasma membrane Na^+^/H^+^ antiporter, plays a significant role in Na^+^ efflux, exporting excess Na^+^ back into the soil [74,75]. Together with *OsSOS1*, other transporters also play important roles to enhance salt tolerance; examples include *OsNHX2*, *OsNHX3*, and *OsNPF*, whose activity increases the sequestration of salt into specific parenchyma cells [76]. The accumulation of Na^+^ from the cytoplasm into the vacuole within cells is an essential mechanism that contributes to plant tolerance [77]. Additionally, the development of root apoplastic barriers, such as the Casparian strip and suberin lamellae, restricts the free movement of Na^+^ through the apoplast, effectively limiting its entry into vascular tissue [78]. NaCl stress induces the excessive production of ROS in rice, including superoxide, H_2_O_2_, and hydroxyl radicals, which disrupt membrane integrity, degrade proteins, and damage nucleic acids [79]. Under NaCl stress, ionic imbalances and osmotic stress disrupt mitochondrial and chloroplast ETC, resulting in ROS overproduction [80]. NaCl stress also disrupts the metabolic pathways of lipids, proteins, and carbohydrates in rice. Salinity causes oxidative damage to membrane lipids through lipid peroxidation, primarily affecting polyunsaturated fatty acids [81]. This peroxidation results in compromised membrane integrity and altered fluidity, which disrupts cellular signaling and ion homeostasis [79]. Salinity stress affects protein synthesis and degradation in rice by altering the expression of stress-responsive proteins, including heat shock proteins (HSPs) and ribosomal proteins [82,83]. Additionally, NaCl induces the unfolding or misfolding of proteins, triggering the expression of chaperons for maintaining the correct protein folding and refolding of denatured proteins, which stabilize protein functions and thus enhance salt tolerance [84]. Salt-tolerant varieties often express higher levels of protective proteins that maintain cellular function during stress conditions. Morphologically, NaCl stress results in reduced shoot and root lengths, lower biomass accumulation, and chlorosis in rice seedlings [85]. Inducing ionic and osmotic stress, the accumulation of Na^+^ and Cl^−^ ions in leaf tissues damages chloroplast structure and alters the photosynthetic machinery reducing the photosystem II efficiency of light capture, which in turn hampers chlorophyll synthesis and carbon assimilation [6,86,87]. The excess Na^+^ interferes with potassium (K^+^) uptake, which is crucial for stomatal regulation and enzyme activation during photosynthesis, due to ionic competition [88]. The germination of rice seeds under NaCl stress is often delayed or inhibited depending on the concentration of NaCl. High salinity creates osmotic stress, reducing water uptake by seeds, which is essential for initiating metabolic activities [89]. In addition, Na^+^ and Cl^−^ ions can accumulate in the embryo and endosperm, disrupting enzyme activity and hormonal regulation [90]. Studies have shown that rice cultivars exhibit varying levels of germination sensitivity to NaCl. Salt-tolerant varieties, such as Pokkali, maintain higher germination rates under salinity stress compared to sensitive varieties, like *IR64* [91]. This tolerance is often associated with efficient osmotic adjustment and ion homeostasis during early development stages. NaCl stress not only affects germination but also significantly hampers seedling growth. Salinity causes osmotic stress, leading to reduced water potential, which impedes cellular expansion and division [6].

In conclusion, HM and salt stresses represent critical environmental constraints that significantly impair rice growth, development, and productivity. Both types of stress induce profound physiological and biochemical disruptions, including oxidative damage, metabolic imbalances, impaired nutrient uptake, and reduced reproductive success. The mechanisms by which rice responds to these stresses, ranging from ion transport regulation and antioxidant defense to metabolic adjustments, highlight the plant’s intrinsic but limited capacity for adaptation. Understanding these responses at the physiological and molecular levels is essential for developing targeted strategies to enhance stress tolerance. Future efforts should focus on integrating physiological insights with genetic improvement, microbial interventions, and sustainable agronomic practices to mitigate the adverse effects of soil contamination and salinization, thereby ensuring food security and crop resilience in increasingly challenging environments. These aspects will be further explored in the following chapters.

## 3. Improving Tolerance to Salt and Heavy Metals Through the Exploitation of Genetic Diversity and Germplasm Resources

**Figure 2:**
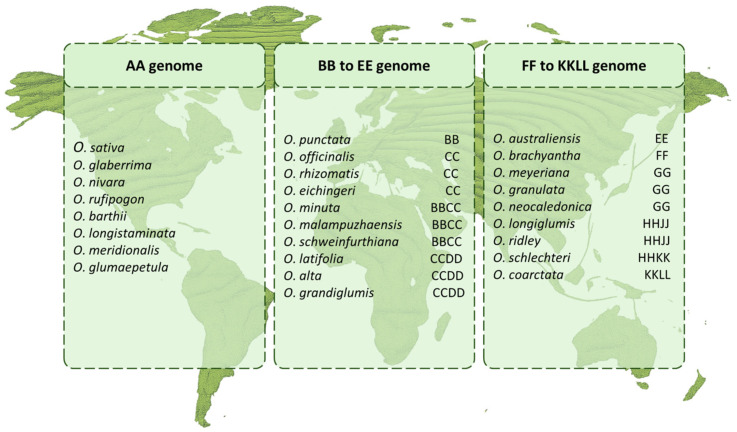
Gene pool of the 27 *Oryza* species (2 cultivated and 25 wild), organized based on their genome type, as proposed by Fornasiero et al., 2025 [93].

*Oryza* genus includes 27 species, organized as 11 different genome types (AA, BB, CC, BBCC, CCDD, EE, FF, GG, KKLL, HHJJ, and HHKK) [92,93]. Of these, only two species are cultivated, namely *Oryza sativa* and *Oryza glaberrima*, both with an AA genome, whose ancestors are recognized in the wild species *O. rufipogon*/*O. nivara* and *O. barthii*, respectively [94]. Among the genetic variability (Figure 2), 10–20% was preserved throughout domestication, but the majority was lost.

Given that wild species are best adapted to harsh environments and suboptimal soil conditions, it is expected that the 25 wild species could provide a significant reservoir of beneficial genes for tolerance to salinity and HMs. Recent advances in next-generation sequencing (NGS) technologies have allowed the sequencing of the wild genomes [92,95,96,97], making available a huge amount of data that might be used to uncover new molecular pathways for stress tolerance or to discover new allelic variants of known genes. Furthermore, collections of wild accessions have been genotyped making available a large set of single nucleotide polymorphisms (SNPs) and indels, describing their great genetic diversity [98,99]. In addition to wild species, over 120,000 *O. sativa* accessions and landraces have been documented [100], with an estimated additional half million landraces in existence [101], constituting a valuable resource for direct exploitation in breeding efforts. Despite the significant number of screening for salt tolerance among diverse collections of rice varieties and landraces [102,103,104], a few of them have been identified as a source of salt tolerance, mainly exploiting the Na^+^ exclusion mechanism in roots, resulting in low Na^+^ accumulation in shoots and a high K^+^/Na^+^ ratio, and utilized for rice improvement [105,106]. The most relevant landraces are Pokkali and Horkuch, coming from the coastal region of Bangladesh, but characterized by a low yield and poor grain quality [107,108,109]. The major salt-tolerance quantitative trait locus (QTL), Saltol, comes from Pokkali, and extensive studies have been performed on allelic variations in the causative gene *HKT1;5* [110,111,112,113,114]. Furthermore, other genes and QTLs have been identified as sources of salt tolerance in these rice landraces, indicating the presence of additional genes employed to lower the Na^+^ concentration in the leaves. For example, the *OsNHX*1 gene from Pokkali has been overexpressed successfully in cultivated rice [115] to enhance salt tolerance, and diverse QTLs and causative genes have been identified in segregating populations with Horkuch as the salt-tolerant parent [106,116]. Investigations of other landrace collections have revealed the existence of salt tolerance pathways beyond Na^+^ exclusion, such as those based on tissue tolerance. Analyzing a collection from the Mekong delta river, in Vietnam, Nguyen and collaborators [117] identified, as a new source of salt tolerance, the landrace Doc Phung, and its gene LOC_Os01g32830—*OsPLGG1*, involved in photorespiration. Additionally, by analyzing different varieties of the African rice *O. glaberrima*, it was shown that salinity-tolerant lines make use of a mechanism of ion homeostasis independent from *HKT1;5* to reduce Na^+^ from shoots [111]. GWAS analyses on landrace or cultivated variety collections have also highlighted the presence of multiple loci, and hence distinct pathways for salt tolerance [118,119,120], paving the way for new sources of tolerance that could be inserted in elite varieties, with a pyramiding approach. Considering HM tolerance, the existence of natural variation for genes associated with Cd accumulation has been reported for *OsCd1*, *OsYSL2*, and *OsHMA3*. Allelic variations in the CDS of *OsCd1* or in the promoter region of CF1 (an allele of *OsYSL2*) and *OsHMA3* regulate the differential accumulation of Cd in grains and have been used to improve elite varieties [121,122,123]. Genetic variation is a major factor affecting also As accumulation in grains, especially in relation to field sites, indicating the possibility of selecting specific cultivars based on the environment [124]. In general, for As, variations in soil and water management have an important impact on the accumulation of grain As, ranging from 100% to 20% [125]. Considering salt stress, taking into account the many resources from wild species, *O. rufipogon* represents one of the best sources of salt-tolerant genes [126,127,128,129] and QTLs [130,131,132] that might be transferred to cultivated elite lines. Among the *Oryza* species sharing the same AA genome structure, crosses between *O.sativa* and, alternatively, *O.rufipogon*, *O.nivara*, or *O. meridionalis* have been reported to increase salt tolerance [133,134,135]. As a consequence, some varieties have been grown in India and Bangladesh (BRRI Dhan 55, DRR Dhan 40, Jaraya, and Chinsurah Nona 2) [136], and some promising introgression lines have been evaluated. Resequencing salt-tolerance related genes has been performed on 103 wild, belonging to *O. nivara* and *O. rufipogon*, and cultivated accessions, showing interesting associations between salt resistance and specific haplotypes, paving the way for future breeding programs [137]. Another promising donor of salt tolerance is *Porteresia coarctata* or *Oryza coarctata,* a halophyte tetraploid wild species (KKLL) growing in the coastal region of Bangladesh and India. This plant takes advantage of a particular mechanism of salinity tolerance: instead of Na^+^ exclusion from xylem vessels and hence from shoots, it accumulates excess Na^+^ in leaf hairs [138]. This is not the only mechanism that *O. coarctata* plants deploy to tolerate severe saline stress. Specific transcriptomic and proteomic responses are activated in *O. coarctata*, suggesting a tonoplast-localized transporter from the NHX family [139]. This suggests Na^+^ vacuolar compartmentalization in the leaves and roots, and the activation of several TFs and metabolic pathways involved in ion homeostasis and tissue tolerance [140]. Among these, the inositol metabolic pathway was recently highlighted as playing a key role in osmolyte regulation in salt stress. Genes involved in inositol and pinitol synthesis (*INO1* and *IMT1*) have been cloned and characterized from this halophyte species [138,141]. Introgression of *PcINO1* by means of transgenesis in cultivated rice and other crops has been shown to confer salt tolerance [142]. Some introgression lines and hybrids are being developed at IRRI [143], overcoming the inter-specific barriers by bridge-crossing with *O. australiensis* [144] or by embryo rescue techniques [145,146], hinting at the possibility of developing new rice-tolerant varieties.

Additional wild genomic resources that have been explored so far come from different surveys that evaluated salt-tolerant *Oryza* species. These studies suggest the presence of distinct metabolic pathways that are specifically activated in these plants, including better control over ionic sodium xylem loading with efficient vacuolar sodium ion sequestration [147], a high tissue tolerance for elevated Na^+^ levels putatively due to vacuolar compartmentalization mediated by specific transporters other than OsNHX1 [105], or enhanced osmoregulation and ion homeostasis via proline pathways [117]. Although the importance of wild genome resources is well recognized, only a few reports exist where genes related to heavy metal resistance have been identified based on analyses of wild rice, landraces, or traditional populations. In a study conducted in 2019 [148], 131 introgression lines (ILs) originating from a cross between *O. nivara* and 93-11 (recurrent parent) were used to identify seven QTLs associated with Cd tolerance. Another study reported a cross between *O. rufipogon*, known to be tolerant to Al in soil, and IR64, which led to a recombinant inbred lines (RILs) population used for QTL mapping [149]. The screening of Single Segment Substitution Lines (SSSLs) represents a technology that helps in the identification of genetic variations associated with a resistance to HMs. For example, screening a library obtained by crossing seven different *Oryza* wild species (AA genome) with the elite variety HJX74 led to the identification of eight QTLs associated with Cd accumulation in grains [150]. SSSLs obtained by crossing *O. glumaepatula* and *O. barthii* with HJX74 as the recipient allowed the development of the SG001 cultivar, which is resilient to Cr stress [151,152]. A comprehensive understanding of the molecular and physiological pathways underlying salt and heavy metal tolerance, combined with the extensive genomic resources that showcase significant genetic variability, may facilitate the identification of causative genes for introduction into elite cultivars. Given that wild relatives frequently introduce undesirable features related to yield and phenology, it is essential to evaluate the most successful technique while also accounting for the challenges posed by interspecific barriers when utilizing species with a non-AA genome organization.

## 4. Mapping and Advanced Breeding to Sustain Tolerance

The accumulation of HMs, toxic non-essential elements, and salt accumulation/tolerance in rice is governed by quantitative traits jointly controlled by multiple genes and the integrated expression of various mechanisms [153,154]. Significant progress has been made by employing forward genetics strategies to identify numerous QTLs and genes associated with HMs and salt tolerance, with GWAS and linkage analysis, which are powerful tools for detecting QTLs associated with complex traits with high accuracy [155]. Advances in DNA sequencing technologies have enabled high-throughput screenings and the identification of several QTLs involved in HM and salt tolerance in rice [156,157,158], and in the following sections a description of newly identified QTLs and candidate genes is provided (summarized in Table 1).

### 4.1. Grain

The two cultivated rice varieties, Japonica and Indica ssp., have great variability in element concentrations. For example, indica rice was found to accumulate higher amounts of Cd in grains, stems, and leaves than japonica rice varieties [164]. A genetic dissection of grain Cd, Pb, As, and Hg toxic HM elements was conducted for 290 indica and 308 japonica accessions through a GWAS study based on element concentrations from three environments allowing the identification of a total of 99 QTLs [159]. Among these, new QTLs for Cd concentration and candidate genes were identified, including *OsNRAMP1* [183] and *OsNRAMP5* [49], encoding natural resistance-associated macrophage proteins, significantly induced by Cd treatment and reducing its accumulation in rice grains and in the end increasing plant tolerance to Cd [184,185]. *OsNRAMP5*, encoding a major transporter for Mn and Cd, was the candidate gene of the major QTL qGMN7.1 for Mn accumulation in rice grain. This QTL was studied in the Chromosome Segment Substitution Line CSSL-qGMN7.1 derived from the cross between cv 93-11 (parent with low grain Mn) with PA64s (parent with high grain Mn), where the QTL region from PA64s was introgressed in the 93-11 background. The analysis of Mn and Cd concentrations in 93-11 vs. CSSL-qGMN7.1 grown in two locations and in pots highlighted that Cd accumulation in CSSL-qGMN7.1 was significantly lower in comparison to cv 93-11, while Mn accumulation was significantly higher in CSSL-qGMN7.1. Indeed, it was observed that sequence variations in *OsNRAMP5* promoter from cv PA64s caused changes in its transcript level in comparison to the *OsNRAMP5* promoter from 93–11 since PA64s-*OsNRAMP5* promoter activity from was stronger than the 93–11-sNRAMP5 promoter [161]. Another locus, Os01g0719300 (qCd1.2) encoding for a sulfate transporter (OsSultr3;6), and its homologous gene of *OsSultr1;1* are involved in Cd and As tolerance and Se accumulation [186]. A combined analysis of LD decay and gene expression identified *OsABCB24*, encoding for an ABC (ATP-binding cassette) transporter as a candidate gene for qCd1-3, a QTL involved in Cd tolerance in rice [164]. The ABC transporters mediate the vacuolar compartmentation of Cd in root tissues in Arabidopsis [187,188], and this gene was significantly expressed at lower levels in high-Cd-accumulative rice accessions than in low-Cd-accumulative accessions. A QTL with a negative effect on Cd accumulation (qCS1) was identified within a BC3F2 population obtained by crossing CSSL10 japonica with 93-11 indica. The QTL co-localized with *OsCS1*, which is allelic to *OsMTP11* (LOC_Os01g62070), a gene expressed in vascular parenchyma cells [165]. Investigations for Pb accumulation in grain [159] identified 12 QTL regions, including as candidates *OsNPF8.1*, *OsHMA6*, and *OsMT2b*, which represent genes affecting heavy metal-element-related traits in previous studies. The gene *OsNPF8.1* (Os01g0142800, encoding for Nitrate Transporter 1/Peptide Transporter 8.1), located in qPb1.1, affects dimethyl arsenate accumulation in grains. *OsHMA6* (Os02g0172600, encoding a heavy metal P-type ATPase) and Os02g0179100, encoding a metal-dependent phosphohydrolase protein, were identified as major candidates for qPb2.2.OsMT2b. Os05g0111300, encoding for the metallothionein gene, and Os06g0542300 encoding for a heavy metal transport/detoxification domain-containing protein, were identified as the most likely candidate genes for qPb5.1 and qPb6.2, respectively. For As concentration, 28 QTLs were identified [159]. Among them, five candidate genes, *OsMTI-3a*, *OsAUX1*, *OsHMA5*, *OsZIP6*, and *OsZIP4*, were identified for qAs1.2, qAs1.6, qAs4.4, qAs5.1, and qAs8.2 QTL regions, respectively. Two candidate genes, Os06g0143700 and Os09g0240500, encoding a sulfate transporter protein, were identified as candidate genes for qAs6.1 and qAs9.1, respectively. The candidate genes Os03g0346800, Os04g0298200, Os05g0382200, and Os08g0117800 encoding cation efflux family proteins were identified for qAs3.2, qAs4.2, qAs5.3, and qAs8.1, respectively. In addition, Os12g0581600 (*OsNRAMP7*) was identified as a major candidate gene for qAs12.4. To identify genetic factors regulating genes involved in As accumulation, a transcriptome-wide association study (TWAS) was conducted using a panel of 273 rice accessions (192 temperate japonica and 49 indica) grown in contaminated soil over two years under flooded and intermittently flooded conditions [160]. The analysis allowed the identification of regulatory regions of several genes involved in transport, detoxification, or stress response, including the cis-eQTLs of AIR2 (arsenic-induced RING finger protein), trans-eQTLs of STR5 (sulfur transferase), and cis-eQTLs of STR8 (sulfur transferase). Classifications based on rice subspecies, genomic sequences, and cis-eQTLs of AIR2 highlighted that the groups clustered in cis-eQTLs and indica varieties had a lower AIR2 expression and As content compared to japonica. This outcome suggests that indica is relatively less likely to be exposed to As risk compared with japonica, and also that the low expression of AIR2 can reduce As accumulation and increase As tolerance in rice [160]. QTLs associated with a reduction in As accumulation in the grain were identified using a diversity panel of 276 rice accessions [166]. From this study, a QTL identified as qGAS12, co-localized with *OsARM1* that encodes a MYB TF, was found to affect As accumulation, while two other QTLs, qGAS8 and qGAS17, were associated with the gene *OsPIP2;7*, encoding for aquaporin, previously identified as being involved in As transport. Overexpression of aquaporin *OsTIP1,2* in rice was found to reduce As accumulation and translocation in rice [189], further supporting the role of aquaporins in As tolerance in rice. Regarding Cu accumulation in grains, a univariate and multivariate QTL analyses for the concentrations of 16 elements in the grains, shoots, and roots of a RILs population grown in different conditions identified a super QTL cluster, where seven grain Cu QTLs were clustered [171]. A candidate genes search in this genomic region uncovered the heavy metal P-type ATPase *OsHMA4*, which had been shown to sequester Cu into root vacuoles and limit Cu accumulation in rice grains [170], suggesting the *OsHMA4* gene may be responsible for this QTL cluster, consequently allowing increased Cu tolerance.

### 4.2. Seedling Stage

An investigation based on a segregating population derived from an indica x japonica cross identified several QTLs for As tolerance at the seedling stage: qAsS2, qAsS5.1, qAsS5.2, qAsS6, qAsS9.1, and qAsS9.2, studied for As content in the shoots were mapped on chromosomes 2, 5, 6, and 9, respectively; qAsR8.1 and qAsR8.2 for As content in roots were mapped on chr 8 and QTL; and qRChlo1 relative chlorophyll content was mapped on chr 1 [162]. For all these QTLs, the As tolerance allele was provided by the indica parent line WTR1, except for qAsS6, where the As tolerance allele was provided by the japonica parent cv Hao-an-nong. Another QTL mapping investigation identified 17 QTLs on different chromosomes, including qCHC-1 and qCHC-3 (chr 1 and chr 3) with candidate genes related to chlorophyll content and qRFW-12 on chr 12 with candidate genes related to root fresh weight [163]. The gene expression analyses of these candidate genes highlighted eight of them: *OsGRL1* (LOC_Os01g13480, Glutaredoxin-like protein 1), *OsDjB1* (LOC_Os0113760, DNAJ family protein), *OsZIP2* (LOC_Os03g29850, metal cation transporter), *OsMATE12* (LOC_Os03g37411, MATE efflux family), *OsTRX29* (LOC_Os12g08730, Thioredoxin M5 family), *OsMADS33* (LOC_Os12g10520, MADS-box transcription factor), *OsABCG29* (LOC_Os12g22110, ABC transporter), and *OsENODL24* (LOC_Os12g26880, plastocyanin-like proteins) as potential key genes for As stress tolerance as they were particularly upregulated in the tolerant line than in susceptible lines [163]. For Cd accumulation, *OsHMA3* for heavy metal ATPase 3 was identified as a candidate gene for qGCdT7 [167]. Phenotypic and physiological evaluations of transgenic rice lines expressing the *OsHMA3* gene, driven by the *OsYSL16* promoter, show it effectively inhibits Cd translocation from the roots to shoots and from the leaves to grains, achieving a reduction in Cd content over 70% and ultimately reducing accumulation in grains [190]. Notably, the expression levels of *OsCAL1* as well *OsIRT1*, *OsIRT2*, *OsNRAMP1*, and *OsHMA2* in the roots of transgenic rice plants were significantly reduced compared to those in WT plants. Another identified Cd-QTL [167] named *CAL1* (Cd accumulation in leaf 1) identified Os02g0629800 as a candidate gene. *CAL1* is expressed preferentially in root exodermis and xylem parenchyma cells and in the leaf sheaths of rice seedlings, and it positively regulates Cd content in leaves and xylem sap [168]. Os02g0629800 encodes a putative defensin precursor, consisting of a cysteine-rich domain and a secretion signal peptide. This suggests that rice accessions with a lower expression of *CAL1* identified through germplasm screening or realized through targeted gene knock-out, should have a phenotype with a lower accumulation and higher tolerance to Cd. In addition, seven QTLs were identified in an IL population in which the donor parent for Cd-tolerance-related genes was *O. nivara*, a wild relative; the identified QTLs were qDRW2, qRRw6, qDSW4, qRSW8, qDTW2, qDTW4, and qRTW8. For the QTLs qDTW4 and qDSW4 on chromosome 4, expression analysis identified as candidate genes a terpene synthase, a cysteine-rich receptor-like protein kinase and a homolog of the carboxypeptidase *OsSCP23* [148]. Finally, for the Cd-tolerant LOC_Os04g27060 containing the *OsAKR1* gene (Os04t0339400), encoding an aldo-keto-reductase, it was demonstrated that Cd exposure increased *OsAKR1* expression [169]. Mutants defective for *OsAKR1* are more susceptible to Cd, thus indicating that this gene represents a good target for Cd tolerance through overexpression, and investigations on allelic diversity for expression levels. Considering salt tolerance, the major Saltol QTL, identified in the salt-tolerant indica landrace Pokkali, accounts for 62–80% of phenotypic variation under salinity stress [179]. It confers salt tolerance to young rice plants by maintaining in the shoots a low Na^+^/K^+^ molar ratio. The *OsHKT1;5* gene located in the Saltol region has been proposed to be the gene responsible for salinity tolerance [180]. *OsHKT1;5* (high-affinity K^+^ transporter) encodes for a xylem-expressed Na^+^ selective transporter and acts by decreasing the Na^+^ content in shoots and maintaining K^+^ homeostasis [72]. Saltol QTL was introgressed from donors IR64-Saltol and FL478 into the temperate japonica genetic background of salt-susceptible European rice varieties using the Marker-Assisted Back-Cross (MABC) approach [181,182]. GWAS combined with linkage analysis revealed additional major QTLs and candidate genes for salt tolerance in japonica rice seedlings. A candidate gene in LOC_Os02g36880, encoding for a NAC TF and negatively regulating salt tolerance at the seedling stage, was identified and its role was confirmed in the CRISPR/Cas9-mediated gene knockout in the japonica Zhonghua11 (ZH11) genetic background [155]. QTL identification for salt-tolerance-related traits in indica rice conducted using a multi-parent advanced generation intercross (MAGIC) population identified a novel QTL (qRRL2) for relative root length and another multi-trait QTL (qSLST1/qRDSW1/qRB1) affecting shoot length, root dry weight, and root biomass under salt treatment. A candidate gene identified at LOC_Os01g66280, encoding a putative transcriptional regulator, was upregulated in the salt-tolerant parent under salt stress, and its expression analysis suggested its involvement in salt tolerance within the multi-trait QTL [177]. Tolerance to mild salinity stress (50 mM NaCl; conductivity of 6 dS m^−1^) in japonica rice investigated in a temperate japonica background by a GWAS mapping study highlighted several QTLs and functions of candidate genes included in calcium signaling and metabolism genes [178].

### 4.3. Reproductive Stage

Only a few studies on salinity tolerance have been conducted for tolerance at the reproductive stage in rice, due to the difficulty of achieving reliable stage-specific phenotyping techniques. In a recent study [173], a BC1F2 mapping population derived from crossing the salt-tolerant variety CSR28 with the salt-sensitive BRRI dhan28 was evaluated for yield components after exposure to the salinity stress of EC 10 dS m^−1^ during the reproductive stage. A total of 15 QTLs were identified, including plant height, panicle length, number of filled and unfilled spikelets, percent of filled spikelets, grain yield, and the Na^+^/K^+^ ratio. Among these, three QTLs, one each for the number of filled spikelets (qNFS10.1), percent of filled spikelets (qPFS10.1), and grain yield (qGY10.1), were mapped at the same position. For all of them, the additive effect was negative, indicating that the tolerant parent CSR28 was responsible for contributing to the QTLs [173]. Another QTL mapping for salinity tolerance was conducted with RILs derived from indica salt-tolerant cv Jarava crossed with salt-sensitive RP Bio226 [174]. Jarava was developed by introgressing genes of agronomic importance from *O. rufipogon* as salinity tolerance at the reproductive stage, broad-spectrum resistance to blast, moderate resistance to brown plant hopper, white-backed plant hopper, and bacterial blight, thus supporting the utilization of wild rice as a source of useful genes. The three major QTLs identified, qSTR-2-qSTR-11 (salinity tolerance rating), qSN-11-qSN-12 (Na^+^ concentration), and qSNK-12.1-qSNK-12.2, located on chromosomes 2, 11, and 12, conferred a yield advantage over the parents under salt stress conditions. Another possible approach for salt stress was identified in the modification of root architecture: the QTL/gene qSOR1 (quantitative trait locus for SOIL SURFACE ROOTING 1) identified in the *O. sativa* japonica ecotype Bulu has a gravitropic effect on the roots and a near isogenic line (NIL) bearing the QTL and subjected to salt stress showed an increase in grain yield with respect to NIL without qSOR1 [175].

### 4.4. Salinity-Tolerance Source from Common Wild Rice (Oryza rufipogon Griff)

Wild rice species of the *Oryza* genus have been recognized for harboring numerous beneficial alleles and genes that could enhance crop yield [191,192]. The exploitation of genetic variability within wild rice is enabling the identification of germplasm resources with low toxic metal and salt contents, QTLs, and promising genes controlling toxic metal and salt contents to improve cultivated rice. Common wild rice (*O. rufipogon*) constitutes the primary gene pool for rice genetic improvement [193] representing an important source of biotic and abiotic stress-response genes [194], including genes for salinity tolerance [195]. This approach was already well addressed in subspecies indica. A total of 87 common wild rice introgression lines (ILs), developed in the Teqing variety background, were evaluated for salt tolerance at the seedling stage, identifying/detecting 15 QTLs related to salt tolerance [130]. Moreover, QTL investigations were performed using a salt-tolerant introgression population, Dongxiang/Ningjing 15 (DJ15), derived from the salt-tolerant wild rice line Dongxiang (*O. rufipogon*) crossed with the *O. sativa* ssp. japonica variety, Ningjing16 (NJ16). Nine QTLs for salt tolerance (qST) at the seedling stage were found, and sequence variant analysis within the QTL regions demonstrated that *SKC1*/*HKT8*/*HKT1;5* and *HAK6* transporters, along with numerous transcriptional factors, were the candidate genes for the salt-tolerant QTL. Among the identified QTLs, qST1.2 and qST6, two QTL with the highest effect for salt tolerance, proved to be more tolerant than the parental lines under salt-stress field conditions, indicating that qST1.2 and qST6 could improve salt tolerance in cultivated rice [132]. In a recent study, whole-genome sequencing was performed for a salt-sensitive ssp. indica cv. KMR3 and *O. rufipogon*-derived IL50-13 to identify introgressed regions from wild rice in the IL50-13 genome for a high yield trait and salinity tolerance at the flowering stage [172]. IL50-13 showed the highest % of germination at 150 mM NaCl, which remained unaffected even under 200 mM NaCl. Within the qGY12.1 (grain yield) QTL, two genes, Os12g0568200 (*OsMT1c*) and Os12g0568500 (*OsMT1Ld*), belonging to metallothionein (MT)-like protein type 1 showed polymorphisms between KMR3 and IL50-13, and transcriptome analysis showed the upregulation of both genes in rice roots in response to oxidative stress.

At the end of this investigation on the available literature related to rice QTLs’ tolerance to toxic elements and candidate genes, it emerges that some gene functions are more frequently identified as related to tolerance in the different tissues/stages considered. In grains and at the seedling stage, the main processes involved are connected to the transport of toxic HMs across cell membranes as heavy metal P-type heavy metal ATPase (HMA) families and ABC transporters. Among them, OsHMA3 is a transport protein involved in chelating Cd into root vacuoles. A represented gene family is also the natural-resistance-associated macrophage protein family (NRAMP) implicated in the accumulation of various HMs, such as *OsNRAMP1* and *OsNRAMP5*, affecting the levels of toxic HM Pb and Cd and Mn in leaves and grains, whereas *OsNRAMP7* is involved in As accumulation in grains. Another category is the cation diffusion facilitator protein family, like the *OsMTP11* gene. Moreover, indications in the involvement of HM tolerance have been observed in processes connected with sulfur transport, the mediation of the vacuolar compartmentation of HMs in roots, as well as the ROS-scavenging ability and enhanced tolerance to HM stress. These are regulated by metallothionein genes (MTs) that can respond to HMs, as well as to environmental stresses, such as drought, salt, and cold [196]. Furthermore, the aldo-keto reductase (AKRs) gene family is frequently identified as being involved in improved tolerance to a variety by scavenging cytotoxic aldehydes; AKRs have been identified to improve Cd tolerance, such as *OsAKR1*. The QTLs and candidate genes related to salt tolerance highlighted various mechanisms acting at the seedling and reproductive stages, such as Na^+^/K^+^ homeostasis in intra/extracellular balance, Ca signaling and metabolism genes, ROS scavenging processes where metallothionein genes (MTs) are involved, and TFs. Moreover, salt tolerance can be improved through changes in root system architecture, in particular, the root growth angle driven by qSOR1; QTL/genes associated with root growth angle caused root development on the soil surface, enabling plants to reduce salt stress. Studies regarding HMs and salt tolerance on wild relative *O. nivara* and common wild rice *O. rufipogon* highlighted new QTLs and candidate genes related to cellular transporters, along with numerous transcriptional factors that could be exploited to improve HMs and salt tolerance, thus supporting wild rice as an important source of new genes and allelic variants for this category of stress tolerance.

## 5. Enhancing Salt and Heavy Metal Tolerance in Rice via Transgenic and Genome Editing Approaches

Recent advances in transcriptomics and metabolomics have provided valuable insights into the complex molecular mechanisms underlying rice responses to HM and salt stress, enabling the identification of novel candidate genes and regulatory pathways for targeted genetic manipulation. Several studies integrating multi-omics analyses have highlighted key transporters, TFs, and metabolic pathways involved in metal detoxification and salt tolerance, offering a foundational resource for developing effective transgenic and genome editing strategies [197,198,199,200]. Transgenic approaches and genome editing techniques have been extensively utilized in order to enhance rice tolerance to HMs and restrict their translocation into grains. Overexpression of multiple genes has demonstrated a significant impact in improving rice HM tolerance. For instance, the overexpression of *OsHMA3*, a P1B-type ATPase, has been shown to enhance vacuolar the sequestration of Cd in root cells, thereby reducing Cd movement to the shoots and grains without affecting essential mineral levels or yield [201,202,203]. Alongside *OsHMA3*, mutations in *OsHMA2* have been found to restrict the translocation of both Zn and Cd from the roots to shoots, thereby lowering the Cd content in grains [204]. Other genes have also demonstrated roles in HM tolerance, such as the overexpression of *OsABCC1* in internode phloem and root cortical cells, which decreased As accumulation in grains by promoting the vacuolar sequestration of As–phytochelatin complexes [205]. Similarly, the overexpression of *OsNIP1* and *OsNIP3*, two aquaporin genes, has been shown to reduce As accumulation by limiting its uptake and transport within the plant [206]. In addition to overexpression approaches, gene silencing through RNA interference (RNAi) has been utilized to influence rice HM tolerance. For example, the RNAi-mediated suppression of *OsPCS1* limits phytochelatin synthesis, thereby reducing Cd accumulation in seeds [207]. In recent years, genome editing, especially via CRISPR/Cas9, has allowed the precise modification of specific genes aiming at reducing HMs uptake and accumulation in rice. Significant advancements involved the target editing of key genes to improve HM tolerance [158,208]. The knockout of *OsNRAMP5*, a principal transporter for Cd and Mn, has been shown to significantly reduce Cd levels in grains without adversely affecting essential minerals uptake or yield [209,210]. Similarly, *OsLCT1*, involved in Cd translocation from the roots to shoots, has been edited to diminish Cd accumulation in grains by restricting its internal movement [211]. The inactivation of a low potassium transporter, *OsHAK1*, has shown to decrease cesium uptake and accumulation [212]. Similarly, the knockout of *OsCCX2*, a putative cation/calcium exchanger involved in Cd accumulation in rice, successfully reduced Cd grain concentration and root/shoot translocation, without negatively affecting yield [213]. Additionally, editing *OsPMEI12* has been associated with enhanced Cd stress resistance, possibly through modifications to cell wall components affecting metal binding and transport [214]. The knockout of *OsZIP2*, implied in Cd root-to-shoot translocation and intervascular transfer, increased Cd allocation in flag leaves but reduced its accumulation in the panicles and grains [215]. Recent CRISPR/Cas9 genome editing efforts have also advanced salt tolerance in rice through targeted mutations of several key genes. Notably, *OsRR22*, a cytokinin-response regulator, has been knocked out to significantly enhance seedling-stage salt tolerance without compromising yield [216]. In another recent study, *OsDSG1*, involved in the ubiquitination pathway, has been edited, resulting in increased plant height, root length, biomass, chlorophyll content, and oxidative stress resistance under saline conditions, maintaining normal growth under non-stress environments [217]. Additionally, the edit of *OsDST*, a stress-responsive TF, has been shown to increase leaf width and reduced stomatal density, contributing to salt tolerance [218]. The mutation of *OsqSOR1*, a homolog of Arabidopsis *DRO1*, resulted in an alteration in root system architecture, promoting shallow rooting and in the end improving salinity tolerance [175]. Furthermore, double mutants generated by editing *Osxlg1* and *Osxlg4* exhibited increased root length and enhanced resistance to salinity stress [219]. Together, these studies highlight a suite of promising gene targets for the development of multi-locus CRISPR strategies to enhance salt and HM tolerance in rice through diverse mechanisms, including hormonal signaling, root morphology modification, and stress-responsive regulation. Despite these advancements, several challenges remain to be addressed. It is essential to evaluate potential off-target effects to ensure precise genome editing, avoiding unintended alterations. Regulatory complexities also pose significant hurdles, as transgenic and genome-edited crops face diverse and often restrictive frameworks worldwide, influencing their market release. Public perception and social acceptance of genetically modified crops can profoundly influence their adoption. Future research should therefore focus on the following different key areas: firstly, multiplex genome editing, simultaneously targeting multiple genes in order to achieve synergistic improvements in stress tolerance; secondly, transgene-free editing technologies, designed to avoid the introduction of foreign DNA and thus address regulatory hurdles and consumer concerns; and thirdly, extensive field trials to validate the agronomic performance of genome-edited rice lines across a diverse range of environmental conditions. Ultimately, it is well-recognized that transgenic and genome editing approaches offer promising strategies to enhance HM and salt tolerance and reduce HM accumulation in rice grains. However, realizing their full potential will require sustained research efforts to ensure food safety and security, along with thoughtful regulatory navigation and proactive public engagement.

## 6. The Role of Microbial Communities in Enhancing Rice Resilience to Inorganic Soil Contaminants

The soil microbiome is a complex and dynamic community whose relevance to plant health and resilience under climate change and environmental stresses is increasingly acknowledged [220]. Plant growth-promoting bacteria (PGPB) have been widely utilized in agriculture to enhance plant productivity through a range of direct and indirect mechanisms, including N fixation, P solubilization, phytohormone production, and biological control [221]. Some PGPB strains also confer tolerance to multiple abiotic stresses, including drought and salinity, by producing 1-aminocyclopropane-1-carboxylate deaminase (ACCD), siderophores, and phytohormones, and by mobilizing essential but otherwise inaccessible nutrients, such as P and K. Halotolerant bacteria are able to survive in saline conditions, while halophilic bacteria require a high salt concentration for growth and have specialized mechanisms to maintain osmotic balance [222,223]. Recently, increasing attention has been paid also to the role of these microorganisms in mitigating the impacts of soil contamination on crop species, including rice. Beneficial microorganisms, including PGPB and mycorrhizal fungi, facilitate tolerance to soil metal contamination, reducing the toxicity of HMs, and modulating their bioavailability in soils [224,225]. PGPB participate in the detoxification processes via biosorption, bioaccumulation, redox transformation, precipitation, and volatilization. Notably, some strains can regulate the expression of metal transporter genes in rice, reducing translocation to edible tissues [226,227]. Root exudates, a complex mixture of organic acids, sugars, amino acids, and secondary metabolites, also play an important role in this process, acting as ecological drivers within the rhizosphere. In this ecosystem, root exudates shape, guide, and influence the microbial community, thereby ensuring optimal conditions for plant growth. It has been shown that root exudates can alter microbial diversity by emulating the signals of quorum-sensing metabolites, thereby acting as an ecological driver of microbial communities in the rhizosphere. In addition, root exudates have been demonstrated to alter soil physical and chemical properties [228]. Microbial activity, conversely, has been shown to modulate the qualitative and quantitative aspects of root exudates affecting root development and providing nutrients for plant growth [229]. The impact of root exudates extends to the biogeochemistry of the rhizosphere and its constituent components. Only a limited amount of total HM content exists in the soil as a soluble component, available for plant uptake. The presence of root exudates has been demonstrated to induce the acidification of the rhizosphere zone, facilitating the conversion of HM free ions from their insoluble and organic forms [230,231,232]. Moreover, it has been well documented that plants are able to secrete certain metal-solubilizing metabolites in the rhizosphere, including organic acids, carboxylates, and certain phytosiderophores, which facilitate HM chelation [233,234]. Microbial modulation of rhizosphere pH and redox potential directly impacts metal speciation, thus influencing their toxicity and root uptake. Flooded paddy soils are characterized by distinct anaerobic microbial ecologies, favoring fermentative bacteria and methanogenic archaea. These conditions drive redox processes that significantly influence organic matter decomposition and nutrient biogeochemical cycling, particularly N and P, and also have profound implications for metal speciation and geochemistry. For example, As mobility and chemical speciation between organic and inorganic forms are strongly modulated by soil redox status [28], and in flooded soils, toxicity and bioavailability are both enhanced [153,235]. Simultaneously, oxygen release from roots forms micro-oxic niches that influence Fe dynamics and methane emissions [236]. Alternate wetting and drying (AWD) practices periodically introduce oxic phases, promoting microbial diversity and enhancing nutrient transformations [237,238].

Moreover, long-term agronomic practices, including crop rotation and fertilization regimes, can substantially impact soil’s buffering capacity, thereby affecting the stability and bioavailability of elements that may act as nutrients or contaminants. Crop rotation has been shown to support microbial diversity and maintain soil fertility, enhancing the resilience of the soil–plant system. In comparison to continuous rice cropping, alternative rotation systems have been demonstrated to markedly enhance surface soil porosity, soil aggregate structure, organic matter, and total N, K, and available P [239]. These strategies are also relevant for the development of sustainable agricultural practices aimed at mitigating the accumulation of soil inorganic contaminants. A recent study in 2025 demonstrated that rotating rice with a Cd-accumulating oilseed rape effectively reduced Cd levels in contaminated farmland [240].

### 6.1. Microbial-Assisted Detoxification and Phytoremediation Mechanisms in Rice for Heavy Metals

The microbial detoxification of HMs relies on a combination of strategies. In biosorption, bacterial species, such as *Bacillus*, use functionalized cell wall groups, carboxyl, hydroxyl, phosphate, to passive immobilize metals, thus limiting their availability to plants [241]. Bioaccumulation, on the other hand, involves an active intracellular sequestration of metals by bacteria like *Rhizobium* and *Enterobacter*, preventing their translocation into plants [242]. Enzymatic reduction/oxidation further detoxifies metals, for example, arsenate and mercuric reductases catalyze the conversion of toxic metal ions into less bioavailable or volatile forms, arsenite (AsIII) is oxidized to arsenate (AsV), while Hg can be methylated and volatilized. These transformations not only reduce toxicity to the host plant but also limit contaminant persistence in soil [227]. These microbial strategies also support phytoremediation approaches, such as phytoextraction and phytostabilization. Rice has been studied in this context, especially when supported by selected microbial inocula [243,244,245,246,247,248,249,250]. These strategies aim either to immobilize contaminants in soil or restrict their translocation beyond the root zone. For example, *Bacillus subtilis* inoculation in rice increased As bioconcentration in shoots and roots while upregulating antioxidant enzyme systems [251]. Another *Bacillus* strain promoted Zn and Cd phytoextraction, increasing plant biomass and reducing the soil bioavailability of these metals [252]. Other examples include co-inoculation strategies, as in Gamalero (2024) [247], where *Bacillus* and arbuscular mycorrhizal fungi improved Cd phytostabilization by reinforcing the root system and restricting metal translocation. In another study, two *Enterobacter* strains enhanced Cd and Ni accumulation in roots while modulating gene expression to alleviate stress [253]. Likewise, other studies documented microbial consortia that improved Ni and Cd accumulation in rice roots while reducing systemic stress responses. The multifaceted role of these microorganisms suggests promising avenues for tailored phytoremediation strategies in contaminated paddy fields [248,249]. A comprehensive table has been included to summarize representative case studies addressing HM stress across the different developmental stages of rice (Table 2).

### 6.2. Microbial Support Against Salinity Stress

In addition to metal-related contamination, salinity represents a major abiotic stress in rice cultivation, particularly in coastal and irrigated regions. Salinity disrupts ionic balance and water uptake, leading to osmotic stress, ion toxicity, and oxidative damage. Certain microbial strains improve rice morphological and physiological parameters under salt stress [277]. Microbial inoculants, particularly halotolerant PGPB, can alleviate salt-induced damage by promoting ionic homeostasis, improving water use efficiency, and reducing oxidative stress. For example, EPS (ExoPolySaccharide) production chelates toxic Na^+^ ions, immobilizes them in the rhizosphere, and facilitates biofilm formation, which contributes to soil aggregation and microbial colonization under salt stress [278]. Proline accumulation in salt-tolerant PGPB acts as an osmoprotectant, maintaining bacterial cell integrity and enhancing stress resilience. Similarly, bacteria-derived ACCD degrades the ethylene precursor 1-aminocyclopropane-1-carboxylate (ACC), reducing ethylene levels in stressed plants and promoting root elongation and nutrient acquisition [279,280]. The phyllosphere microbial consortia *Bacillus marisflavi* and *Pantoea stewartia*, isolated from mangrove plants, demonstrated a higher content of metabolites associated with salt tolerance and nutrient preservation, such as L-glutamate, aspartic acid and betaine metabolites [281]. The application of *Pseudomonas stutzeri* and *Klebsiella pneumoniae*, isolated from the rice rhizosphere, has been shown to significantly improve seedling growth under saline conditions, through the production of IAA (Indole-3-acetic acid), nitrogenase, ammonia, and siderophores [282]. Salinity also impairs N and S assimilation by increasing Cl^−^ uptake, making N-fixing halotolerant PGPB even more critical [283]. PGPB-mediated salt stress mitigation also includes the production of antioxidant enzymes, like SOD, CAT, and glutathione reductase GSH, which counteract oxidative damage triggered by salt-induced ROS [284,285]. Halotolerant endophytes, such as *Curtobacterium oceanosedimentum*, *Enterobacter ludwigii*, and *Bacillus cereus*, have demonstrated the ability to boost shoot and root growth, enhance antioxidant responses (e.g., GSH production), and increase soluble sugar levels in rice plants under salt stress [286]. For instance, MDA, a lipid peroxidation product, dramatically increases under NaCl exposure, but is reduced in the presence of inoculated bacteria, indicating lower oxidative stress levels in treated rice plants [285]. Recent genomic insights reveal that PGPB strains, such as *Bacillus* NMTD17 and *Enterobacter cancerogenus* (JY65), activate a suite of stress-responsive genes in rice, including *DegU*/*DegS*, *SodA*/*SodB*, *OpuAC*/*OpuD*, and *HPII*, involved in stress response, osmolyte transport, and antioxidant enzyme production. These bacteria also upregulate *OsYUCCA1* and *OsPIN1*, key genes in auxin biosynthesis and transport, contributing to improved root development [243,287]. Enhanced expression of biofilm-related genes (e.g., *bssSR*, *YjbE*, *wcaD*) has been observed in rice inoculated with *Curtobacterium* and *Enterobacter* strains [286]. Table 3 summarizes the studies addressing salt stress across different developmental stages of rice.

PGPB represents promising tools for sustainable rice cultivation in contaminated or salt-affected soils. Their effectiveness is often enhanced when native strains adapted to specific environments are used, underscoring the importance of local screening and characterization. However, challenges remain in ensuring microbial persistence under field conditions, optimizing consortia compatibility, and scaling applications in diverse agroecosystems. Future work should also focus on the combined effects of multiple stressors, such as salinity and metal toxicity, and how microbial consortia can be tailored to confer broad-spectrum resilience in rice. The integration with ecological approaches, which also consider soil microbiome and agronomic management, is essential for a comprehensive understanding of rice responses to HM and salinity stress in real-word cultivation scenarios.

## 7. Conclusions and Perspectives

The persistent increase in the population and the consequent rise in demand for healthy foods are exerting pressure on available resources, resulting in high land and water consumption, as well as environmental pollution. Rice cultivation, particularly the submergence technique, is subjected to environmental stresses, such as elevated HM concentrations and heightened soil salinity, consequences of contemporary rice-cultivating practices. These toxic elements have strong resistance to biodegradation, posing serious risks to crop production and human health. This review synthesizes the findings from recent studies on molecular sciences, thereby enhancing our understanding of the genetic mechanisms underlying resistance in the context of pollution caused by HMs and high salt concentrations. In the current era, the necessity to expedite the identification of genes associated with the response and tolerance to these external pressures is mounting. The identification of such genes is crucial in order to facilitate the development of new genotypes that exhibit enhanced tolerance to HMs and salt. The adoption of such varieties would contribute to the mitigation of production losses incurred due to soil contaminants and concurrently reduce adverse effects on food security and human health. Genetic diversity for improving tolerance to salinity and heavy metal stress is significantly contributed to by wild species, which are better adapted than cultivated species to extreme environments. Valuable allelic variants and molecular pathways underlying stress responses have been revealed by advances in genome sequencing and genotyping. In addition to the extensive collection of *O. sativa* landraces and accessions, these wild genetic resources possess considerable potential for developing stress-tolerant cultivars through breeding methodologies. Indeed, the research conducted on *O. nivara* and *O. rufipogon* has led to the identification of novel QTLs and candidate genes, thereby underscoring the significance of wild germplasm as a repository of adaptive traits. It must be taken into account that transferring genes and/or QTL from wild species could be challenging considering the introgression of undesirable traits in linkage and, in the case of wild species not belonging to the AA genome species, the inter-species fertility barrier must be overcome. In this case, biotechnological approaches, such as editing or transgenic techniques, might be helpful. A multitude of QTLs associated with HM and salt tolerance have been identified through GWAS, linkage analyses, DNA sequencing, and validation through functional genomics approaches. The literature concerning QTLs and candidate genes on this topic emphasizes key molecular functions that are recurrently implicated across developmental stages and tissues. A central role played in HM detoxification was ascribed to genes related to metal transport, including HMAs, ABC transporters, and NRAMP family members through vacuolar sequestration and membrane transport. Additional mechanisms include sulfur metabolism, ROS scavenging, metallothioneins, and aldo-keto reductase. In the context of salt tolerance, factors, such as Na^+^/K^+^ homeostasis, Ca signaling, and root system architecture, particularly root growth angle, have been identified as critical traits. Increasing evidence underscores the role of non-coding RNAs, including microRNAs (miRNAs) and long non-coding RNAs (lncRNAs), as critical modulators of stress-responsive gene expression, often through the refinement of transcriptional and post-transcriptional networks, thus emerging as promising targets for improving stress tolerance. In accordance with these findings, genome editing technologies, particularly CRISPR/Cas9, have facilitated the precise manipulation of stress-related genes, thereby providing new opportunities for breeding. For instance, the knockout of *OsNRAMP5* has effectively reduced Cd accumulation in grains without affecting the yield or essential mineral uptake. In light of the modulation of genes associated with hormonal signaling, root morphology, and stress responses, it can be posited that multi-locus editing strategies hold promise in enhancing rice tolerance to multiple abiotic stresses. The integration of these approaches has emerged as a promising strategy for cultivating rice cultivars that exhibit resilience and adaptation to challenging environments. Rice tolerance to high concentrations of metals and salt stress can be fostered through the use of plant PGPB, which support vegetative growth and confer resilience to stress through both direct and indirect mechanisms. The induction of tolerance by PGPB has been thoroughly investigated in relation to the modulation of plant gene expression, as well as through the production of bioactive compounds. Furthermore, complementary strategies, including soil detoxification and pollutant immobilization, have been investigated. Moreover, the application of microbiome engineering through PGPB and rhizosphere modulation could offer other sustainable methods to enhance tolerance through nutrient cycling, pollutant immobilization, and systemic resistance. The use of synthetic communities (SynCom), engineered microbial communities designed with specific strains, revealed in recent years another research topic that deserves further investigation. In summary, the intricate nature of rice responses to HM and salt stress necessitates an interdisciplinary approach that combines diverse scientific disciplines. The integration of agronomic practices, advanced breeding techniques, biotechnological tools, and microbiome-based strategies is not only desirable but essential to develop effective and sustainable solutions. Insights from soil science elucidates soil physicochemical characteristics and contaminant dynamics should be integrated with plant physiology and molecular biology, which uncover intrinsic plant adaptive mechanisms, and microbial ecology, which reveals the critical roles of rhizosphere and endophytic microbial communities. This interdisciplinary approach will enable a comprehensive understanding of the complex soil–plant–microbe nexus and facilitate the development of innovative, sustainable strategies for enhancing rice resilience under field conditions. Future efforts should continue to promote such cross-disciplinary collaborations combining the latest technologies and biology systems to deal with the many factors involved in plant abiotic stress tolerance.

## Figures and Tables

**Table 1:** QTLs for heavy metal and salt tolerance in rice.

Element	Dev Stage/ Organ	QTL Name	Candidate Genes	Start (bp)	End (bp)	Lead SNP/Marker	Known Gene	Gene Function	Genetic Material	References
As	Grain	qAs1.2	Os01g0200700	5,478,545	5,479,749		*OsMTI-3a*	similar to metallothionein-like protein type 3 (MT- 3) (MWMT3)	598 rice germoplasms include 290 Xian (*O. sativa* ssp indica) and 308 Geng (*O. sativa* ssp japonica) rice	[159]
		qAs1.4	Os01g0595201	23,324,460	23,325,125			heavy metal-associated domain containing protein, expressed		
		qAs1.6	Os01g0856500	36,998,338	37,004,643		*OsAUX1*	auxin transporter, primary root and root hair elongation, Cd stress response		
		qAs2.3	Os02g0510600	18,252,570	18,256,034			heavy metal-associated transport/detoxification protein		
		qAs2.4	Os02g0818900	35,130,674	35,131,877			heavy metal metal-associated domain transport/detoxification protein domain		
		qAs3.2	Os03g0346800	12,954,637	12,959,631			cation efflux family proteins, putative, expressed		
		qAs3.5	Os03g0819400	34,386,215	34,388,174			heavy metal-associated domain transport/detoxification protein domain		
		qAs4.2	Os04g0298200	13,176,753	13,183,064			cation efflux family proteins, putative, expressed		
		qAs4.3	Os04g0533900	26,684,584	26,687,711			heavy metal-associated domain transport/detoxification protein domain		
		qAs4.4	Os04g0556000	27,829,841	27,835,533		*OsHMA5*	heavy metal P-type ATPase, xylem loading of Cu		
		qAs5.1	Os05g0164800	3,807,974	3,810,780		*OsZIP6*	transition metal, ion transporter		
		qAs5.3	Os05g0382200	18,475,523	18,479,481		NaT, *OsCHX11*	Na^+^ transporter		
		qAs6.1	Os06g0143700	2,292,664	2,298,802		*OsSultr3*	sulfate transporter protein		
		qAs8.1	Os08g0117800	981,210	984,101		*OsCHX08*	cation/H^+^ exchanger domain containing protein		
		qAs8.2	Os08g0207500	6,267,823	6,270,904		*OsZIP4*	similar to Zn transporter ZIP1		
		qAs8.2	Os08g0205400	6,159,123	6,161,383			heavy metal-associated domain containing protein		
		qAs8.2	Os08g0205500	6,161,997	6,163,054			HMA domain containing protein		
		qAs9.1	Os09g0240500	3,073,972	3,092,832		*OsSultr4*	sulfate transporter protein		
		qAs12.4	Os12g0581600	24,119,866	24,123,724		*OsNRAMP7*			
		cis-eQTLs AIR2	Os11g0572500	19,200,000	25,800,000		*AIR2*	similar to sulfate transporter 4.1, chloroplast precursor (AST82)	rice core collection of 273 accessions: 192 temperate japonica, 19 tropical japonica, 49 indica, 8 aus, 3 admixture, and 2 aromatic varieties	[160]
		trans-eQTLs STR5 (chr.01)	supplementary table Lee et al., 2022 [160]	25,400,000	33,600,000		*STR5*	sulfur transferase		
		cis-eQTLs STR8	Os02g0157600	3,000,000	3,100,000		*STR8*	sulfur transferase- arsenate As(V) reductase, As(V) As tolerant		
		qGAS8; qGAS17	Os09g0541000	21,310,132	21,311,479		*OsPIP2;7*	aquaporin involved in As transport	276 accessions (*O. sativa* ssp. indica)	[161]
		qGAS12	Os05t0442400	21,655,380	21,654,183		*OsARM1*	MYB transcription factor responsible for As translocation		
							*OsPT3*	P transporter		
		qGAS1	LOC_Os01g55500	31,917,858	33,339,115			nucleobase-ascorbate transporter		
			LOC_Os01g55600	32,032,686	32,035,011			nitrate transporter		
			LOC_Os01g55610	32,038,018	32,040,378					
			LOC_Os01g56050	32,270,054	32,272,147			MATE protein		
	Seedling stage	qRChlo1 Rel. Chl. content	LOC_Os01g67720 LOC_Os01g67580 LOC_Os01g67770	39,282,883	39,420,824			ABC1 family domain containing protein, putative, expressed, multidrug-resistance-associated protein, putative	BRILs (indica WTR1 and japonica cv. Hao-an-nong)	[162]
		qAsR8.1 As content root	LOC_Os02g13520 LOC_Os02g11760 LOC_Os02g09720 LOC_Os02g09150 LOC_Os02g13560 LOC_Os02g10760 LOC_Os02g10070 LOC_Os02g10690 LOC_Os02g08500 LOC_Os02g09650	6,057,678	6,057,678			*OsIAA7*—auxin-responsive Aux/IAA gene family member, expressed		
		qAsR8.2 As content root		7,854,002	7,854,002					
		qAsS2 As content shoot		4,342,883	7,277,487					
		qAsS5.1 As content shoot		10,886,331	14,643,984					
		qAsS5.2 As content shoot		15,469,279	16,808,642					
		qAsS6 As content shoot		400,753	2,025,629					
		qAsS9.1 As content shoot		18,366,555	19,208,050					
		qAsS9.2 As content shoot		20,587,039	21,348,882					
		qCHC-1 Chl. content	LOC_Os01g13480	RM10458	RM5459		*OsGRL1*	glutaredoxin-like protein 1	120 doubled haploid (CNDH) lines *(O. sativa* ssp. indica cv. Cheongcheong and japonica cv. Nagdong)	[163]
		qCHC-1 Chl. content	LOC_Os01g13760	RM10458	RM5459		*OsDjB1*	DNAJ family protein		
		qCHC-3 Chl. content	LOC_Os03g29850	RM6931	RM6266		*OsZIP2*	metal cation transporter		
		qCHC-3 Chl. content	LOC_Os03g37411	RM6931	RM6266		*OsMATE12*	MATE efflux family		
		qRFW-12 Root fresh weight	LOC_Os12g08730	RM247	RM1261		*OsTRX29*	thioredoxin M5 family		
		qRFW-12 Root fresh weight	LOC_Os12g10520	RM247	RM1261		*OsMADS33*	MADS-box transcription factor		
		qRFW-12 Root fresh weight	LOC_Os12g22110	RM247	RM1261		*OsABCG29*	ABC transporter		
		qRFW-12 Root fresh weight	LOC_Os12g26880	RM247	RM1261		*OsENODL24*	plastocyanin-like proteins		
Cd	Grain	qCd1.2	Os01g0719300	29,987,911	29,994,075		*OsSultr3;6*	similar to sulfate transporter 3.1	598 rice germoplasm include 290 Xian (*O. sativa* ssp. indica) and 308 Geng (O. *sativa* ssp. japonica) rice	[159]
		qCd7.2	Os07g0257200	8,871,643	8,878,905		*OsNRAMP5*	Mn/Cd transporter, Mn/Cd uptake		
		qCd7.2	Os07g0258400	8,966,025	8,970,880		*OsNRAMP1*			
		qCd1-3	Os01g0911300	43,219,290	43,355,290	rs1_43287290	*OsABCB24*	ABC transporter B family member 24	338 mainly indica rice accessions grown in Cd-contaminated soils with different Cd contents	[164]
		qCS1	LOC_Os01g62070	R35728	R36152		*OsCS1/OsMTP11*	manganese transporter	BC3F2 (CSSL10 × cv. *O. sativa* ssp. indica 93-11)	[165]
		qGCD7	LOC_Os12g41090	25,444,742	25,446,274			CBL-interacting protein kinase.	276 accessions *O. sativa* ssp. indica	[166]
		qGCD9	LOC_Os02g35000	20,994,405	20,997,117			chaperone protein dnaJ 10		
		qGCD13	LOC_Os04g33920	20,543,471	20,543,934					
		qGCD14	LOC_Os06g41450	24,844,743	24,843,829			vacuole domain containing protein		
		qGCD17	LOC_Os03g60720	34,511,279	34,507,597					
	Seedling stage	qGCdT7	LOC_Os07g12900	6,062,000	16,811,000		*OsHMA3*	heavy metal ATPase 3—root-to-shoot Cd translocation	SSSLs (elite indica cv. Huajingxian 74 and indica cv. BG367)	[167]
		CAL1—Cd accumulation in leaf 1	Os02g0629800	11,389,878	25,871,290		*CAL1*	similar to defensin precursor *CAL1* specifically mediates Cd efflux	119 DH, CSSL, 3651 BC3F3 (indica Cd-over-accumulating cv. Tainan1—TN1 and cv. Chunjiang06—CJ06)	[168]
		qDRW2; qDTW2				Bin202			ILs (*O. nivara* x indica 93-11)	[148,169]
		qRRW6				Bin581				
		qRSW8; qRTW8	-			Bin774				
		qDSW4; qDTW4	LOC_Os04g27060	16,005,150	16,001,505	Bin429	*OsAKR1*	aldo-cheto-reductase		
		qDSW4; qDTW4	LOC_Os04g27190	16,068,256	16,057,969	Bin429		terpene synthase		
		qDSW4; qDTW4	LOC_Os04g25060	33,641,298	33,642,277	Bin429		cyst-rich receptor-like protein kinase		
		qDSW4; qDTW4	LOC_Os04g25560	14,819,280	14,813,270	Bin429		carboxypeptidase homologue *OsSCP23*		
Cd/Mn	Grain	qGMN7.1 Grain Mn accumulation	LOC_Os07g15370	8,286,947	9,313,202		*OsNRAMP5*	major transporter for Mn and Cd	132 RILs/fine mapping on CSSL-qGMN7.1 (indica cv. 93-11—low grain Mn × indica-like variety PA64s- with maternal origin of japonica, high grain Mn)	[161]
Cu	Grain	QTL Cluster 2	LOC_Os02g10290	4,987,000	5,798,000		*OsHMA4*	heavy metal-transporting type ATPase	LT-RILs (Tropical japonica cv. Lemont × indica cv. TeQing)	[170,171]
Hg	Grain	qHg3.1	Os03g0161200	3,271,150	3,276,878		*OsSultr3*	similar to sulfate transporter	598 rice germoplasms include 290 Xian (*O. sativa* ssp. indica) and 308 Geng (*O. sativa* ssp japonica) rice	[168]
Pb	Grain	qPb1.1	Os01g0142800	2,294,904	2,298,329		*OsNPF8.1*	putative peptide transporter, translocation of dimethylarsinate to rice grain	598 rice germoplasms include 290 Xian (*O. sativa* ssp. indica) and 308 Geng (*O. sativa* ssp. japonica) rice	[159]
		qPb2.2	Os02g0172600	3,950,459	3,955,971		*OsHMA6*	similar to heavy metal ATPase		
		qPb2.2	Os02g0179100	4,384,188	4,387,935			heavy metal accumulation, metal-dependent phosphohydrolase		
		qPb5.1	Os05g0111300	605,868	606,764		*OsMT2b*	metallothionein gene		
		qPb6.2	Os06g0542300	20,404,356	20,405,495			heavy metal accumulation–transport–detox protein		
Na^+^	Flowering stage	qGY2.1	Os02g0187100	4,831,212	4,833,985			similar to cyclase	IL50-13 a salt-tolerant IL, cv. notified as Chinsurah Nona 2 (Gosaba 6) (derived by crossing a salt-sensitive (*O. sativa* ssp. indica) KMR3 ((Karnataka Mandya Restorer 3) × *O. rufipogon*) IL50-13 IL derived from KMR3 × *O. rufipogon* after 4 backcrosses with KMR3	[172]
		qGY2.1	Os02g0194400	5,259,822	5,266,512			similar to receptor-like kinase; leucine-rich repeat receptor-like kinase, Cd stress response		
		qGY2.1	Os02g0294700	11,209,135	11,211,943			topoisomerase II-associated protein PAT1 domain containing protein		
		qGY11	Os11g0606800	23,415,625	23,418,653					
		qGY11	Os11g0618800	24,096,264	24,097,002			hypothetical conserved gene		
		qGY12.1	Os12g0568200	23,383,189	23,384,177			metallothionein-like protein type 1		
		qGY12.1	Os12g0568500	23,390,501	23,391,407		*Os1MT1Ld*	metallothionein-like protein type 1		
		qGY12.1	Os12g0566800	23,302,307	23,306,305		*OsMT1c*	ion channel regulatory protein, UNC-93 domain containing protein		
		qGY12.1	Os12g0564800	23,167,167	23,171,951			NB-ARC domain containing protein		
		qGY12.1	Os12g0565100	23,182,563	23,188,498			NB-ARC domain containing protein; NB-ARC domain containing protein		
		qGY12.1	Os12g0566200	23,271,514	23,272,915			conserved hypothetical protein		
		qGY12.1	Os12g0566300	23,275,214	23,279,087			subunit A of the heteromeric ATP-citrate lyase, disease resistance		
		qGY12.1	Os12g0566500	23,290,875	23,292,674					
	Reproductive stage	qNFS10.1 N. filled spikelets		18,730,000	19,378,174	K_id10005402-K_id10006100			624 BC1F2 mapping population derived from CSR28 (salt-tolerant Indian cv.) × BRRI dhan28 (salt-sensitive indica Bangladeshi cv.)	[173]
		qPFS10.1% filled spikelets								
		qGY10.1								
		qNFS10.1 N. filled spikelets								
		qSN-11 Na^+^ concentration		RM26622	RM21				184 RILs (salt sensitive RP Bio226-indica x salt-tolerant Jarava—indica)	[174]
		qSN-12 Na^+^ concentration		RM17	RM28587					
	Roots	qSOR1	Os07g0614400	25,309,034	25,311,637		*qSOR1*	surface roots system	*O. sativa* japonica ecotype Bulu	[175]
	Seedling stage	qRRL2 Rel. root length	LOC_Os02g36880	21,864,234	24,239,570		LOC_Os02g36880		189 RILs (CD—salt-sensitive × WD20342—salt-tolerant) 295 japonica rice materials gathered from Chinese provinces and varieties from Japan, Russia, and Korea	[176]
		qRRDW2 Rel. root dry weight	LOC_Os02g37000 LOC_Os02g37080					NAC transcription factor, negatively regulated salt tolerance at the seedling stage		
		qRRL2 Rel. root length		25,860,000	27,880,000				MAGIC (indica four parents, SAGC-08 (A), HHZ5SAL9-Y3-Y1(B), BP1976B-2-3-7-TB-1-1(C), PR33282-B-8-1-11-1-1 (D), and 221 DC1)	[177]
		qSLST1/qRDSW1/qRB1 shoot length under salt treatment, rel. dry shoot weight, rel. biomass (multi-trait QTL)	LOC_Os01g66280	38,180,000	38,570,000			putative transcriptional regulator	MAGIC (indica four parents: SAGC-08 (A), HHZ5SAL9-Y3-Y1(B), BP1976B-2-3-7-TB-1-1(C), PR33282-B-8-1-11-1-1 (D), and 221 DC1)	
		q02_02 Rel. root dry weight	LOC_Os02g18690	10,897,172	10,901,913		*OsBURP04*	BURP domain containing protein	231 *O. sativa* ssp. japonica accessions	[178]
		q02_02 Rel. root dry weight	LOC_Os02g18880	11,015,828	11,017,808		*OsCBL7*	calcineurin B, putative, expressed		
		q02_02 Rel. root dry weight	LOC_Os02g18930	11,057,896	11,059,975		*OsCBL8*	calcineurin B, putative, expressed		
		q02_02 Rel. root dry weight	LOC_Os02g21009	12,432,287	12,448,557		*OsCAX1c*	Na^+^/Ca^2+^ exchanger protein, putative		
		q02_06 Leaf area	LOC_Os02g36880	22,258,833	22,260,681		*OsNAC1*	no apical meristem protein, putative		
		q02_06 Leaf area	LOC_Os02g36974	22,333,281	22,337,713		*GF14E*	14-3-3 protein, putative, expressed		
		q03_02 Rel. Leaf area	LOC_Os03g27280	15,628,100	15,632,465		*SAPK1*	CAMK_like.19 Ca^2+^/calmodulin dependent protein kinases, expressed		
		q03_03 Leaf area	LOC_Os03g27960	16,061,151	16,065,169		*OsCAX2*	Na^+^/Ca^2+^ exchanger protein, putative		
		q03_03 Leaf area	LOC_Os03g28120	16,163,989	16,167,050		*OsKAT1*	K^+^ channel protein, putative, expressed		
		q06_02 Rel. Leaf area	LOC_Os06g10880	5,677,080	5,682,126		*OsABF2*	bZIP transcription factor, putative		
		qST1.1 Salinity Tolerance	Os01g0917400 Os01g0926700 Os01g0926800 Os01g0932500	40,006,067	40,907,438	40,300,000	*OsHAK6*	high-affinity K^+^ transporter 6 (Os01g0932500)	RILs F2, 4 n = 103 (DJ15 (salt-tolerant IL derived from Dongxiang (*O. rufipogon*) × Ningjing 16 (NJ16)) × cv. Koshihikari japonica salt-sensitive)	[132]
		qST1.2^DJ15^ Salinity Tolerance	Os01g0276800 Os01g0279100 Os01g0281200 Os01g0293000 Os01g0295900 Os01g0297700 Os01g0298301 Os01g0298400 Os01g0298500 Os01g0299300 Os01g0302500 Os01g0305900 Os01g0307500 Os01g0310500	9,874,379	11,666,398	10,600,000	*OsSKC1/HKT8/HKT1;5*	Protein kinase, catalytic domain containing protein (Os01g0307500)		
		qST6^DJ15^ Salinity Tolerance	Os06g0635700 Os06g0636100 Os06g0636600 Os06g0636700 Os06g0636800 Os06g0637800 Os06g0639100 Os06g0639200 Os06g0639500 Os06g0640201 Os06g0640800 Os06t0641066 Os06g0641575 Os06g0642550 Os06g0643000 Os06g0643500 Os06g0644600 Os06g0645100	25,600,000	26,324,093	25,600,000				
Na^+^/K^+^	Reproductive stage	qSTR-2 Salinity tolerance rating		RM110	RM423				184 RILs (salt-sensitive RP Bio226 indica × salt-tolerant Jarava indica)	[174]
		qSTR-11 Salinity tolerance rating		RM286	RM3717					
		qSNK-12.1 Na^+^/K^+^ concentration		RM17	RM28587				184 RILs (salt-sensitive RP Bio226 indica × salt-tolerant Jarava—indica)	[174]
		qSNK-12.2 Na^+^/K^+^ concentration								
	Seedling stage	Saltol QTL	Os01g0307500	10,690,930	12,591,394		*OsHKT1;5 (SKC1)*	high-affinity K^+^ transporter, low Na^+^ uptake, high K^+^ uptake and Na^+^/K^+^ homeostasis in shoots	F8 RILs/BC3F4 NILs (ssp. indica landrace Pokkali × salt-sensitive ssp. indica cv. IR29)	[179,180,181,182]

Table shows, for each element, the phenological phase evaluated, QTL name, the associated candidate genes, the genomic position, the potential function, the segregating population or collection in which the QTL was identified, and the parental lines used for analysis.

**Table 2:** Microbial enhancement of rice tolerance to HM stress.

Element	Microorganism	Experimental Setup	Effect on Plants	References
Al	SynCom, including 2 strains each of *Paenibacillus*, *Lysinibacillus*, and *Burkholderia*, three strains of *Bacillus*, and one strain each of *Leucobacter*, *Pseudomonas* and *Rhodococcus*	Pot and field	Improved rice Al resistance and alleviated P deficiency. Reduced root growth angle for P acquisition in topsoil.	[254]
	*Serratia marcescens* (MO4), *Enterobacter asburiae* (MO5), *Pseudomonas veronii* (R4), and *Pseudomonas protegens* (CHAO)	Pot	Promoted plant growth, increased plant height, mitigated Al toxicity reducing its bioavailability through Al^3+^ chelation, and reduced plant uptake by enhancing EPS secretion.	[255]
	*Bacillus subtilis*	Pot	Promoted plant growth and productivity in terms of plant height, chlorophyll content, tiller number, panicle number, grain yield, root growth, and root biomass.	[256]
As	*Bacillus subtilis* IU31	Pot	Increased bioconcentration and bioaccumulation factors in shoot and roots. Improved plant by health restoring normal levels of GST, CAT, GSH, and H_2_O_2_. Contributed to As detoxification, thus increasing its uptake.	[251]
	*Acinetobacter indicus*	Pot	Acceleration of Fe, Cu, and Ni uptake; activation of SOD, CAT, guaiacol peroxidase, glutathione peroxidase, glutathione-s-transferase, reduction in oxidative stress, MDA, and methylglyoxal generation.	[257]
	*Cupriavidus taiwanensis* KKU2500–3 and *Pseudomonas stutzeri* 4.44	Seedling stage	Promoted plant growth, reduced toxicity and accumulation in roots and shoots, increased enzymatic and non-enzymatic antioxidant compounds, and reduced oxidative stress.	[258]
	*Pantoea dispersa*	Hydroponic	Increased shoot and root length, fresh and dry weight, seedling vigor index, total sugar content, total protein content, chlorophyll, and reduced MDA and As concentration.	[259]
Cd	*Pseudomonas koreensis*	Hydroponic and pot	Promoted plant growth; reduced Cd shoot, root, and grain concentration; upregulated the synthesis of phenylpropanoids and flavonoids; increased the activity of antioxidant enzymes, proline, and GSH; reduced MDA and H_2_O_2_ levels.	[251]
	*Pseudomonas* sp. 4N2 and *Bacillus* sp. TB1	Hydroponic	Promoted plant growth, increased antioxidant activity, reduced in Cd transfer from roots to shoots, bacterial immobilization, and root phytostabilization.	[260]
	*Rhodopseudomonas palustris* SC06	Pot	Shaped bacterial community; reduced bioavailable Cd; upregulated sugar, organic acids, and antioxidant enzymes in rice roots; reduced Cd uptake in rice seedlings; reduced Cd concentration in roots, stems, leaves, and grains; improved photosynthetic efficiency in leaves.	[261]
	*Herbaspirillim* sp. and *Bacillus cereus*	Hydroponic	Herbaspirillum reduced Cd uptake; Bacillus promoted Cd uptake. Effects on endophytic bacterial community in roots.	[248]
	*Cupriavidus taiwanensis* KKU2500–3	Pot	Reduced Cd translocation to stems, leaves, and grain; reduced Cd concentration in grains; increased in leaves pigments.	[262]
	*Cupriavidus metallidurans* CML2	Pot	Plant growth promotion (IAA production and phosphorus solubilization, siderophore production). Increase in root length and decrease in Cd bioaccumulation in seedlings and translocation rates.	[263]
	*Enterobacter tabaci* 4M9	Seedling stage	Promoted plant growth, reduced oxidative stress and electrolyte leakage, CAT, and SOF.	[264]
Cr	*Staphylococcus aureus* L.	Seedling stage	Transformation to a less toxic form of Cr, reduction in plant Cr uptake, and enhanced chlorophyll content.	[265]
	*Staphylococcus aureus* L.	Pot	Plant growth and yield promotion; increased SPAD values, total chlorophyll, and carotenoids; reduced MDA, H_2_O_2_, and electrolyte leakage in shoots; increased POX, CAT, APX, and SOD activity; enhanced macro- and micronutrients in shoots; and reduced Cr concentration in roots, shoots, and grains.	[266]
Cr+Cd	*Lysinibacillus* sp. OR-15	Pot	Promoted plant growth and reproduction. Alleviated Cr and Cd stress. Fe plaques formed around roots increased aboveground Cr and Cd concentrations (immobilization), but they were reduced in the stems and seeds.	[267]
Cu	*Micrococcus yunnanensis* GKSM13	Seedling stage	Increased plant length and seed vigor index, reduced Cu stress, increased SOD, CAT, APOX, and GPOX activity, and reduced MDA concentration and DPPH inhibition.	[268]
Fe	*Bacillus cereus* GGBSU-1, *Klebsiella variicola* AUH-KAM-9 and *Proteus mirabilis* TL14-1	Pot	Increase in bioavailability of P and other micronutrients, reducing the nutrient limitations occurring in ferruginous soils and limiting Fe toxicity by Fe chelation. Positive effects also on soil–microbiota colonization.	[269]
	*Bacillus cereus* MZ157036, *Staphylococcus coagulans* MZ157032, *Pseudomonas aeruginosa MZ157041*, *B. paramycoides* MZ157031, *Ps. aeruginosa* MZ157040, *Ps. aeruginosa* MZ157039, *B. tequilensis* MN715782, and *B. wiedmannii* MN715783	Field	Plant growth and nutrient uptake promotion. Increase in N, P, and K uptake. Increase in Fe uptake in roots, shoots, and grains.	[270]
Mn	*Rhodopseudomonas palustris* TLS12, VNS19, VNS32, VNS62 and VNW95, and *Rhodopseudomonas harwoodiae* TLW42	Pot and field	Promoted plant growth and production and soil fertility; reduced Mn plant concentration.	[271]
Ni + Cd	*Enterobacter ludwigii* SAK5 and *Exiguobacterium indicum* SA22	Hydroponic	Promoted plant growth, increased chlorophyll content, increased root accumulation of both Cd and Ni, upregulated metal stress-responsive genes, and protected rice from heavy metal hyperaccumulation.	[253]
	*Pseudomonas* sp., *Chryseobacterium* sp., and *Enterobacter* sp.	Pot	Promoted plant growth, increased antioxidant enzyme activity in seedlings, and mitigated oxidative damage.	[272]
Pb	*Bacillus altitudinis* IHBT-705	Pot	Improved shoot length, root length, total roots, chlorophyll content, antioxidant enzyme activity, and decreased Pb concentration in rice plants.	[273]
Se	*Priestia* sp. LWS1	Pot	Increased rice biomass and Se concentration.	[274]
Zn +Cd	*Bacillus* sp. ZC3-2-1	Pot	Decreased Zn and Cd concentrations in soil and increased phytoextraction and immobilization. Increased rice biomass. No change in Zn and Cd content per biomass unit. No negative effect on crop food safety.	[252]
Zn	*Bacillus* sp. SH-10 and *Bacillus cereus* SH-17	Field	Improved yield and grain Zn content alone and in combination with chemical fertilization. Increase in chlorophyll content and Zn-requiring enzymes.	[275]
	*S. marcescens* FA-4	Pot and field	Promoted plant growth, yield, and grain Zn content; increased SOD and CAT enzyme activity.	[276]

The table reports the HMs involved, the microbial strains used, the experimental setup, and the observed effects on growth, physiology, or yield-related traits.

**Table 3:** Microbial enhancement of rice tolerance to salt stress.

Element	Microorganism	Experimental Setup	Effect on Plants	References
NaCl	*Bacillus* NMTD17, *Bacillus* GBSW22	Pot	Increased relative abundance of rhizobacterial species; strong biofilm formation up to 16% of NaCl concentration. Decreased levels of ROS Upregulation of: *DegU* and *DegS* genes (stress mitigating response) *SodA* and *SodB* (superoxide dismutase production) *OpuAC* and *OpuD* genes (betaine metabolites) *HPII* gene (catalase regulation) *ComA* gene (quorum-sensing regulation) Under high saline conditions (200 mmol), NMTD17 promoted: Highest vigor index (VI) in rice seedlings, Increased root morphological parameters (volume, area, length, diameter and tips)	[243]
NaCl	*Bacillus subtilis* BRAM_G1, *Bacillus subtilis* BRAM_G2, *Mesobacillus subterraneus* BRAM_Y2, *Brevibacillus parabrevis* BRAM_Y3	Pot, 5% salt	Increased chlorophyll concentration, seed weight, grain filling, plant height and reproductive parameters	[277]
NaCl	*Bacillus marisflavi*, *Pantoea stewartia*	Pot	Increased shoot and root length Increased L-glutamate, aspartic acid, betaine metabolites, L-lysine, soluble sugars, and K^+^ Decreases MDA and Na^+^ Promoted salt tolerance of *C. islandicus*	[281]
NaCl	*Curtobacterium oceanosedimentum*, *Curtobacterium luteum*, *Enterobacter ludwigii*, *E. tabaci*, *Bacillus cereus*, *Micrococcus yunnanensis*	Pot, 150 mM NaCl	Increased shoot and root length, biomass, and fresh and dry weight Increased ABA content, GSH amount, and soluble sugars Upregulated the *OsYUCCA1* gene (IAA biosynthesis) and *OsPIN1* gene (auxins production)	[286]
NaCl	*Enterobacter cancerogenus* (JY65)	48-well plate	Increased weight, height and root length of plants Increased GSH, ascorbic acid, APX, SOD, POD, CAT, and K^+^ Decreased Na^+^, ROS, and MDA Increased biofilm formation (*bssSR*), exopolysaccharide producing protein YjbE, and colonic acid biosynthesis-related genes (*wcaD*, *wzbc*, *etkp*) Genes related to PGP traits identified: IAA production, polyamines, N2-fixation, siderophores, volatile organic compound, antimicrobial compound, and phosphate solubilization	[287]
NaCl	*P. alhagi* NX-11	Hydroponic, 100 mM NaCl	Increased production of antioxidant enzymes SOD, POD, and CAT on the 7th day after salt stress treatment Increased EPSs and MDA content	[285]

The table reports the microbial strains used, the experimental setup, and the observed effects on rice growth, physiology, or yield-related traits.

## Data Availability

No new data were created or analyzed in this study. Data sharing is not applicable to this article.

## Abbreviations

The following abbreviations are used in this manuscript:

ACDD	1-aminocyclopropane-1-carboxylate deaminase
AKR	Aldo-keto reductase
As	Arsenic
APX	Ascorbate peroxidase
ATP	Adenosine triphosphate
B	Boron
BC1F2	Back-cross F2 generation
BC3F2	Third back-cross F2 generation
bZIP	Basic leucine zipper
Ca	Calcium
CAT	Catalase
Cd	Cadmium
Cl	Chlorine
CO_2_	Carbon dioxide
Cr	Chromium
CRISPR/Cas9	Clustered Regularly Interspaced Short Palindromic Repeats and CRISPR-associated protein 9
Cu	Copper
EPSs	ExoPolySaccharides
eQTL	Expression QTL
ETC	Electron transport chain
F0	Parental generation
F1	First generation, from 2 parental lines
Fe	Iron
GSH	Glutathione reductase
GWAS	Genome wide association study
H_2_O_2_	Hydrogen peroxide
Hg	Mercury
HMs	Heavy metals
IAA	Indole acetic acid
IL	Introgression line
K	Potassium
MAGIC	Multi-parent Advanced Generation InterCross
MDA	Malondialdehyde
Mg	Magnesium
Mn	Manganese
Mo	Molybdenum
N	Nitrogen
Na	Sodium
NAC	NAM, ATAF1/2, CUC2
NaCl	Sodium chloride
NIL	Near isogenic line
P	Phosphorus
Pb	Lead
PGPB	Plant growth-promoting bacteria
QTL	Quantitative trait locus
RIL	Recombinant inbred line
RNAi	RNA interference
ROS	Reactive oxygen species
Se	Selenium
SNP	Single-nucleotide polymorphism
SOD	Super-oxide dismutase
SSSLs	Single-segment substitution lines
SynCom	Synthetic community
TF	Transcription factor
Tl	Thallium
Zn	Zinc
